# 

**Published:** 1881-05

**Authors:** 


### BOOKS AND PAMPHLETS RECEIVED.

The Principles and Practice of Surgery. By D. Hayes Agnew, M.D., LL.D. Vol. II. Philadelphia: J. B. Lippincott & Co.; 1881.

Medical Diagnosis with Special Reference to Practical Medicine. By J. M. DaCosta, M.D. Philadelphia: J. B. Lippincott & Co.; 1881.

A Practical Treatise on Diseases of the Skin. By Louis A. Duhring, M.D. Philadelphia: J. B. Lippincott & Co.; 1881.

A Manual on Diseases of the Eye and Ear, for the use of Students and Practitioners. By W. F. Mittendorf, M.D. New York: G. P. Putnam's Sons; 1881.

Syphilis and Marriage. By Alfred Fournier. Translated by P. Albert Morrow, M.D. New York: D. Appleton & Co.; 1881.

On the Construction, Organization, and General Arrangements of Hospitals for the Insane. By Thomas S. Kirkbride, M.D., LL.D. Philadelphia: J. B. Lippincott & Co.; 1881.

How to See with the Microscope. By J. Edwards Smith, M.D. Chicago: Duncan Brothers; 1880.

An Elementary Treatise on Practical Chemistry and Qualitative Inorganic Analysis. By Frank Clowes, D. SC. Philadelphia: Henry C. Lea's Son & Co.; 1881.

On the Use of Cold Pack, followed by Massage, in the Treatment of Anæmia. By Mary Putnam Jacobi, M.D., and Victoria A. White, M.D. New York: G. P. Putnam's Sons; 1880.

The Heart and its Function. D. Appleton & Co.; 1881.

Manual of the Physical Diagnosis of Diseases of the Heart. By Arthur Ernest Sansom, M.D. Philadelphia: Presley Blakiston; 1881.

A Guide to the Clinical Examination of Patients and the Diagnosis of Disease. By Richard Hagen, M.D. Boericke & Tafel, New York and Philadelphia; 1881. Translated by G. E. Gramm, M.D.

An Index of Comparative Therapeutics. By Samuel O. L. Potter, M.D. Chicago: Duncan Brothers; 1880.

Aids to Diagnosis—Part II; Physical. By J. C. Thorowgood, M.D. New New York: G. P. Putnam's Sons; 1881.

"Doctor, What Shall I Eat?"—A Handbook of Diet in Disease. By C. Gatchell, M.D. Milwaukee; 1880.

A German-English Dictionary of Words and Terms used in Medicine and its Cognate Sciences. By Tancourt Barnes, M.D. Philadelphia: Presley Blakiston; 1881.

The Bacteria. By Dr. Antoine Magnin. Translated by George M. Sternberg, M.D. Boston: Little, Brown & Co.; 1880.

Spectacles, and How to Choose Them. By C. H. Vilas, M.A., M.D. Chicago: Duncan Brothers; 1881.

Aphorisms in Fracture. By Richard O. Cowling, A.M., M.D. Louisville, Ky.: John P. Morton & Co.; 1881.

On "Kerion Celsi;" A Variety of Tinea Tonsurans. By I. E. Atkinson, M.D. Reprinted from the Archives of Dermatology, Vol. VII, No. 1; January, 1881.

Seventh Annual Report of the Superintendent of the Cincinnati Sanitarium for the year ending November 30, 1880.

Erysipelas of the Larynx. By Wm. Porter, A.M., M.D. St. Louis: Re printed from Archives of Laryngology, Vol. I, No. 4, December, 1880.

Higher Education of Medical Men, and its Influence on the Profession and the Public. By F. D. Lente, M.D.

Colorado for Invalids By S. Edwin Solly. Reprinted from "New Colorado and the Santa Fe Trail," by permission of Harper Bros.; 1880.

A Conspectus of Three Different Forms of Cardiac Trophic Disease. By Roswell Park, M.D. Reprint from the "Southern Clinic."

Proceedings of the Convention of Druggists, and of the First Meeting of the Illinois Pharmaceutical Association, held at Springfield, December 7 and 8, 1880.

Transactions of the Eleventh Annual Session of the Medical Society of Virginia; held in Danville, October 19–21, 1880.

---

ZYMOTIC DISEASES IN NEW YORK.—Three thousand eight hundred and ninety-four deaths reported in 1880, were referred to zymotic diseases, influenced or aggravated by defective plumbing.

SOCIETY MEETINGS.

Chicago Medical Society—Mondays, May 2 and 16.
West Chicago Medical Society—Mondays, May 9 and 23.
Biological Society—Wednesday, May 4.

CLINICS.

MONDAY.

Eye and Ear Infirmary—2 p. m., Ophthalmological, by Prof. Holmes; 3 p. m., Otological, by Prof. Jones.
Mercy Hospital—2 p. m., Surgical, by Prof. Andrews.
Rush Medical College—2 p. m., Dermatological and Venereal, by Prof. Hyde.
Woman's Medical College—2 p. m., Dermatological and Venereal, by Prof. Maynard; 3 p. m., Diseases of the Chest, Prof. Ingals.

TUESDAY.

Cook County Hospital—2 to 4 p. m., Medical and Surgical Clinics.
Mercy Hospital—2 p. m., Medical, by Prof. Quine.

WEDNESDAY.

Chicago Medical College—2 p. m., Eye and Ear, by Prof. Jones.
Rush Medical College—2 p. m., Medical, by Dr. Bridge; 3 p. m., Ophthalmological and Otological, by Prof. Holmes; 3:30 to 4:30 p. m., Diseases of the Chest, by Dr. E. Fletcher Ingals.

THURSDAY.

Chicago Medical College—2 p. m., Gynæcological, by Prof. Jenks.
Rush Medical College—2 p. m., Diseases of Children, by Dr. Knox; 3 p. m., Diseases of the Nervous System, by Prof. Lyman.
Eye and Ear Infirmary—2 p. m., Ophthalmological, by Dr. Hotz.
Woman's Medical College—3 p. m., Surgical, by Prof. Owens.

FRIDAY.

Cook County Hospital—2 to 4 p. m., Medical and Surgical Clinics.
Mercy Hospital—2 p. m., Medical, by Prof. Davis.

SATURDAY.

Rush Medical College—2 p. m., Surgical, by Prof. Gunn; 3 p. m., Orthopœdic, by Prof. Owens.
Chicago Medical College—2 p. m., Surgical, by Prof. Isham; 3 p. m., Neurological, by Prof. Jewell.
Woman's Medical College—11 a. m., Ophthalmological, by Prof. Montgomery; 2 p. m., Gynæcological, by Prof. Fitch.
Daily Clinics, from 2 to 4 p. m., at the Central Free Dispensary, and at the South Side Dispensary.

